# Differential Impact of a Multicomponent Goals-of-Care Program in Patients with Hematologic and Solid Malignancies

**DOI:** 10.3390/cancers15051507

**Published:** 2023-02-28

**Authors:** David Hui, Sairah Ahmed, Nico Nortje, Marina George, Clark R. Andersen, Kaycee Wilson, Diana Urbauer, Christopher Flowers, Eduardo Bruera

**Affiliations:** 1 Department of Palliative, Rehabilitation and Integrative Medicine, MD Anderson Cancer Center, Houston, TX 77030, USA; 2 Department of Lymphoma and Myeloma, MD Anderson Cancer Center, Houston, TX 77030, USA; 3 Department of Stem Cell Transplant and Cellular Therapy, MD Anderson Cancer Center, Houston, TX 77030, USA; 4 Department of Critical Care Medicine, MD Anderson Cancer Center, Houston, TX 77030, USA; 5 Department of Hospital Medicine, Division of Internal Medicine, Houston, TX 77030, USA; 6 Department of Biostatistics, MD Anderson Cancer Center, Houston, TX 77030, USA; 7 Department of Inpatient Analytics, MD Anderson Cancer Center, Houston, TX 77030, USA; 8 Division of Cancer Medicine, MD Anderson Cancer Center, Houston, TX 77030, USA

**Keywords:** advance care planning, communication, hematologic neoplasms, intensive care units, palliative care, quality of health care

## Abstract

**Simple Summary:**

Goals-of-care discussions can help patients to understand their illness and to share their values and treatment preferences with their clinicians. Here, we compared the impact of an institution-wide goals-of-care program on intensive care unit (ICU) mortality between patients with hematologic malignancies and solid tumors. We found that patients with blood cancers had improved goals-of-care documentation, but no change in ICU mortality; in contrast, patients with solid tumors had lesser improvement in goals-of-care documentation, but significantly lower ICU mortality. These findings highlight the need to overcome other barriers to improve care for patients with hematologic malignancies.

**Abstract:**

We recently reported that an interdisciplinary multicomponent goals-of-care (myGOC) program was associated with an improvement in goals-of-care (GOC) documentation and hospital outcomes; however, it is unclear if the benefit was uniform between patients with hematologic malignancies and solid tumors. In this retrospective cohort study, we compared the change in hospital outcomes and GOC documentation before and after myGOC program implementation between patients with hematologic malignancies and solid tumors. We examined the change in outcomes in consecutive medical inpatients before (May 2019–December 2019) and after (May 2020–December 2020) implementation of the myGOC program. The primary outcome was intensive care unit (ICU) mortality. Secondary outcomes included GOC documentation. In total, 5036 (43.4%) patients with hematologic malignancies and 6563 (56.6%) with solid tumors were included. Patients with hematologic malignancies had no significant change in ICU mortality between 2019 and 2020 (26.4% vs. 28.3%), while patients with solid tumors had a significant reduction (32.6% vs. 18.8%) with a significant between-group difference (OR 2.29, 95% CI 1.35, 3.88; *p* = 0.004). GOC documentation improved significantly in both groups, with greater changes observed in the hematologic group. Despite greater GOC documentation in the hematologic group, ICU mortality only improved in patients with solid tumors.

## 1. Introduction

Patients with hematologic malignancies such as lymphoma, leukemia, and myeloma often experience substantial symptom burden as their disease progresses [1,2,3]. Compared with patients with solid tumors, these patients often have higher rates of emergency room visits, hospitalizations, and intensive care unit (ICU) admissions, and lower rates of advance care plans, do-not-resuscitate (DNR) orders, specialist palliative care referral, and hospice utilization as they approach the last months of life [4,5]. This pattern of end-of-life care may be explained by several unique aspects of hematologic oncology, including a wider array of therapeutic options, greater prognostic uncertainty, and different attitudes and beliefs toward end-of-life care between hematologic and solid tumor oncologists [6,7].

Goals-of-care discussions represent an important clinical tool to improve patient care. These discussions are semi-structured communication interventions to enhance the patients’ prognostic awareness and acceptance, to elicit their values and preferences towards end-of-life care, and to formulate a personalized care plan [8,9,10,11]. Several studies have found that goals-of-care discussions are associated with a reduction in intensive treatments at the end of life [12,13,14,15]. However, only a handful of studies have specifically examined the impact of goals-of-care discussions in patients with hematologic malignancies [16].

Although randomized controlled trials have be used to examine structured goals-of-care interventions, the assessment of complex multimodal communication interventions in the real world requires other designs. We recently conducted a study to examine hospital outcomes before and after implementation of a system-wide multicomponent interdisciplinary goals-of-care program (myGOC) among medical inpatients at our comprehensive cancer center. After propensity score adjustment, we found a significant reduction in ICU mortality rate (−6.3%), ICU length of stay (−1.4 days), and overall hospital mortality (−0.9%) after program implementation [17]. However, it was unclear if patients with hematologic malignancies benefited to the same extent as patients with solid tumors. Thus, we conducted this secondary analysis to compare the change in ICU mortality rate before and after implementation of myGOC program between patients with hematologic malignancies and solid tumors.

## 2. Methods

### 2.1. Study Design

This is pre-planned secondary analysis with a retrospective cohort design to assess the impact of our myGOC program on hospitalization outcomes comparing patients with solid tumors and those with hematologic malignancies. The eligibility criteria have been reported previously [17]. Briefly, we included consecutive medical inpatients admitted to the University of Texas MD Anderson Cancer Center between 1 May 2019 and 31 December 2019 (pre-myGOC implementation period) and between 1 May 2020 and 31 December 2020 (post-myGOC implementation period). The Institutional Review Board at MD Anderson Cancer Center approved this study and provided a waiver for informed consent. 

### 2.2. Study Intervention

myGOC was developed in response to the COVID-19 pandemic and started in March 2020. This program was implemented on a system-wide basis, with involvement of hospital leadership, medical oncology, surgical oncology, palliative care, hospitalist service, emergency medicine, intensivists, social work, ethics, case management, chaplaincy, and nursing. The six key components of this program have been described elsewhere [17]. Briefly, they included (1) risk stratification to identify patients at high risk of hospital mortality based on established prognostic factors, (2) oncologist-led goals-of-care discussions with patients identified as high risk followed by documentation of these conversations, (3) same day follow up by departmental leaders for patients identified as high risk but without documented goals-of-care discussions, (4) specialist palliative care team provided goals-of-care training for oncology teams and supported the deployment of goals-of-care interdisciplinary rapid response team for complex situations, (5) longitudinal monitoring of myGOC operation and hospital metrics by an institutional goals-of-care committee, and (6) active involvement of hospital leadership and emphasizing goals-of-care as an institutional priority.

### 2.3. Data Collection

We collected patient demographics on admission, such as age, sex, race, ethnicity, cancer diagnosis, primary admitting service, admission type, COVID-19 infection, and Sequential Organ Failure Assessment (SOFA) score from the electronic health record. SOFA score is a validated prognostic score initially developed in patients admitted to the ICU [18]. It consists of eight variables. The total score ranges from 0 to 24. Initial SOFA scores of 0–1, 2–3, 4–5, 6–7, 8–9, 10–11, 12–14, and >14 correspond to a mortality rate of 0, 6.4%, 20.2%, 21.5%, 33.3%, 50%, 95.2%, and 95.2%, respectively [19]. The increase in SOFA scores over time also indicates increased mortality. The area under the receiver operating characteristic curve has been reported to be between 0.61 and 0.88 [18]. 

Patients admitted under lymphoma and myeloma, leukemia, and stem cell transplant services were coded as having hematologic malignancies. For this analysis, we excluded patients with benign tumors (e.g., ductal carcinoma in situ and neurofibromatosis).

The primary study outcome was ICU mortality, which was defined as the number of patients who died in the ICU divided by the number of patients discharged from the ICU (including deaths). Secondary outcomes included ICU length of stay, ICU admission, hospital mortality, and hospital length of stay. We also assessed the proportion and timing of completion in relation to the index hospital admission for the following documents: goals-of-care discussions using a note template, do-not-resuscitate (DNR) orders, living will (LW, a signed legal document indicating the patient’s preference for life sustaining measures), medical power of attorney (MPOA, a signed legal document identifying the healthcare surrogate(s) in the event of decisional incapacity), and out-of-hospital DNR orders (OOHDNR). 

### 2.4. Statistical Analysis

This is a secondary analysis of a study examining GOC. We estimated that we had 80% power to detect a 5% reduction in mortality using a two-tailed test at a 5% significance level with a baseline ICU mortality of 28% and approximately 600 medical ICU patients in each time period.

We summarized patient characteristics and outcomes with descriptive statistics, including proportions, 95% confidence intervals (CIs), means, standard deviations, medians, and interquartile ranges (IQRs).

For patients with multiple admissions within each cohort time period (i.e., 2019 and 2020), we randomly selected a single hospitalization per patient to keep observations independent. There was minimal overlap in the two cohort time periods (<1%). Thus, they were treated as independent hospitalizations in statistical analyses.

Baseline demographic and clinical characteristics were summarized by cancer group (solid vs. hematological) and time period (2019 vs. 2000) as means with standard deviations or percentages with standard deviations. Following modeling by linear or logistic analysis of variance, contrasts were used to assess differences between time periods separately by cancer group, as well as differences between cancer types between time periods. 

Continuous outcomes were modeled by multivariable analysis of variance with relation to cancer group (solid vs. hematological) and time period (2019 vs. 2000), while controlling for covariates including age, gender, race/ethnicity, hospital admission type (elective, emergency, urgent), and first SOFA score. Contrasts with Hommel-adjusted *p*-values were used to assess differences between time periods separately by cancer group, as well as differences between cancer types in differences between time periods. Binary outcomes, including the primary outcome of ICU mortality, were similarly modeled by logistic regression. The count of ICU admissions during the same hospital stay was similarly modeled by negative binomial regression. 

The Kaplan–Meier method was used to summarize overall survival from ICU admission by cancer group and time period, with differences assessed by the log-rank test. Overall survival from ICU admission was modeled using a parametric accelerated failure time model with log-logistic distribution (selected per Akaike information criteria among Weibull, exponential, Gaussian, logistic, log-normal, and log-logistic distributions, and verified by residual plot overlaid on the distribution) (Figure S1); a Cox proportional hazard model was ruled out owing to violation of the proportionality of hazards assumption.

We also examined the association between ACP note completion status (not completed by the end of index hospitalization; completed before index hospitalization; and completed only during index hospitalization) and ICU mortality rate with logistic regression in both patients with solid tumor and those with hematologic malignancies. 

Because the prognosis of patients with solid tumors may differ significantly from those with hematologic malignancies, we conducted a sensitivity analysis by adjusting for clinician-predicted survival provided at the time of ICU admission. Prognosis was estimated by the ICU attending physician based on five categories: >1 year, 6 months to 1 year, 3–6 months and acute issues likely reversible, 3–6 months and acute complications likely irreversible, and prognosis < 3 months [17,20]. Because this score was only available in the post-pandemic period, the analysis was limited to ICU patients in 2020. 

R statistical software (R version 4.2.2, The R Foundation for Statistical Computing, Vienna, Austria) was used for all statistical analyses, and a 95% level of statistical confidence was assumed.

## 3. Results

### 3.1. Patient Characteristics

This study included 11,599 medical patients over the 16-month study period, with 1253 (10.8%) ICU admissions. In total, 5036 (43.4%) patients had hematologic malignancies and 6563 (56.6%) had solid tumors. Patient characteristics are shown in Table 1. The mean age was 60.8 (SD 14.6), 5034 (43.4%) were female, and 7218 (62.2%) were white. Moreover, 1750 (34.7%) of the admissions for patients with hematologic malignancies were elective admissions, in contrast to 572 (8.7%) of the admission for patients with solid tumors. The mean SOFA score was 2.8 (2.5).

### 3.2. Change in ICU Outcomes in Medical Patients with Hematologic Malignancies and Solid Tumors

Table 2 shows that patients with hematologic malignancies had no significant change in ICU mortality between 2019 and 2020 (26.4% vs. 28.3%; odds ratio (OR) 1.10, 95% confidence interval (CI) 0.75, 1.61); in contrast, patients with solid tumors had a significant reduction (32.6% vs. 18.8%; OR 0.48, 95% CI 0.33, 0.69). The between-group difference in change in ICU mortality was statistically significant (OR 2.29, 95% CI 1.35, 3.88; *p* = 0.004). 

ICU length of stay was also significantly lower between 2019 and 2020 in the patients with solid tumors, but not in patients with hematologic malignancies. However, the between-group difference was not statistically significant (Table 2).

The rate of ICU admission, number of ICU admissions, and overall survival from ICU admission did not change significantly between 2019 and 2020 in both groups. 

### 3.3. Change in Hospital Outcomes in Medical Patients with Hematologic Malignancies and Solid Tumors

Overall hospital mortality was significantly lower between 2019 and 2020 in patients with solid tumors (6.3% vs. 4.9%; OR 0.77, 95% CI 0.63, 0.93), but not in patients with hematologic malignancies (4.7% vs. 4.9%; OR 1.06; 95% CI 0.83, 1.35) (Table 2). The length of stay did not differ.

### 3.4. Goals-of-Care Documentation

DNR order during the index hospitalization increased significantly in both groups between 2019 and 2020 (solid tumor: 16% vs. 22%; hematologic malignancy: 7% vs. 12%; Table 3). The timing of DNR order establishment also shifted earlier for both groups, particularly for patients with hematologic malignancies.

Similarly, the frequency of ACP note documentation increased and the timing of documentation from admission decreased significantly in both groups (solid tumor: 15% vs. 48%; hematologic malignancy: 5% vs. 53%; Table 3). The magnitude of change in the hematologic malignancy group was significantly greater. Although there were some improvements in LW, MPOA, and OOHDNR, the changes did not differ significantly between the two groups, with the exception of the timing of OOHDNR, which became much earlier among patients with hematologic malignancies. 

### 3.5. Association between ICU Mortality Rate and ACP Completion Status

Among patients who had their ACP note completed during the hospital visit, we observed a significant reduction in ICU mortality after myGOC implementation in both the solid tumor and hematologic malignancy groups (Table 4). In contrast, we did not detect a statistically significant difference in ICU mortality between 2019 and 2020 when ACP documentation was either not completed or completed prior to the hospitalization, despite a trend towards a reduction in the solid tumor group. 

### 3.6. Sensitivity Analysis

Accounting for prognosis based on the ICU priority score, the hematologic malignancy group had significantly higher rates of ICU mortality, ICU length of stay, and shorter overall survival than the solid tumor group (Table 5). These results were similar to the unadjusted data (Table 2).

## 4. Discussion

In this pre-planned secondary analysis, ICU mortality reduced significantly post-intervention in patients with solid tumors, but not those with hematologic malignancies, despite greater improvement in GOC documentation in the post-intervention period in the hematologic group. Taken together, our findings highlight that patients with hematological malignancies remain at risk for more intensive treatments at the end of life. This differential finding suggests that GOC discussions were likely necessary but insufficient to impact hospital outcomes. Further studies are needed to overcome modifiable barriers to quality end-of-life care for patients with hematologic malignancies.

We previously reported that ICU and hospital outcomes improved significantly post-intervention [17]. This secondary analysis revealed that much of these improvements were limited to patients with solid tumors. These changes were consistent with our pre-specified program goal and the mechanism of action of the multicomponent system-wide intervention [17,21]. We found that patients who had ACP note completed during the index hospitalization, but not before or never, had a particularly significant reduction in ICU mortality, suggesting that the timing of GOC discussions matters. Indeed, enhanced prognostic discussions and acceptance, facilitated decision making regarding cancer treatments at the end-of-life [22,23], referrals to specialist palliative care [24,25], psychological support and spiritual care [26], thoughtful discharge and care planning, and timely establishment of DNR status in patients with high mortality risk, coupled with intensive monitoring and leadership support, all likely contributed to these improved outcomes. Of note, this study examined outcomes in all hospitalized patients, while the myGOC intervention focused on patients with a poor prognosis. We expect the magnitude of change to be even greater if only patients with advanced cancer were included.

Although GOC discussions may also reduce ICU admissions, we only observed a reduction in ICU mortality rate. Potential explanations may include (1) greater focus of the myGOC intervention on ICU patients, including deployment of rapid-response team [27], (2) patients and families requiring time to make difficult decisions, and (3) their preference for time-limited trials rather than forgoing ICU care altogether.

In contrast to patients with solid tumors, we were surprised to find that patients with hematologic malignancies had no improvement in ICU and hospital outcomes post-intervention. One potential explanation is that many patients with hematological malignancies were in the ICU for acute potentially reversible complications, while patients with solid tumors were largely there as an end-of-life event. In the pre-intervention period, ICU and overall hospital mortality were lower and the length of stay was longer among patients with hematologic malignancies relative to those with solid tumors, consistent with the literature that these patients had slightly better outcomes and needed longer term support. However, the median survival from ICU admission to death was less than 2 weeks for both groups, underscoring how close to the end of life these patients were. Our sensitivity analysis adjusting for prognosis also revealed similar findings. The lack of change in hospital outcomes in the hematologic group post-intervention could not be explained by the lack of effort in GOC documentation. Other potential reasons that could contribute to this finding may include the variable quality of GOC discussions [16,28], greater prognostic uncertainty [29], continual emphasis of cancer treatments and curability [6], lack of or delayed referral for palliative care and hospice care [4,30,31], and lower rates of DNR orders among the hematologic group compared with the solid tumor group. Further research is needed to examine these issues. 

The differential impact of GOC documentation and hospital outcomes provides useful insights into how we can improve the quality of end-of-life care. The clinical and significant increase in frequency and earlier GOC documentation in patients with hematologic malignancy were encouraging. In the pre-intervention period, GOC documentation was generally lower and delayed in the hematologic group compared with the solid tumor group. 

However, we observed a substantial improvement in the post-intervention period, surpassing the solid tumor group for some metrics. The lack of an improvement in hospital outcomes despite the dramatic improvement in GOC documentations may lead to questions regarding the effectiveness of GOC interventions; however, the substantial changes in outcomes in patients with solid tumors, the underlying mechanisms, and the greater literature would support that GOC interventions are useful [12,13,14,15,17]. Rather, our study findings underscore that an improvement in GOC documentation may not be enough to change hospital outcomes in patients with hematologic malignancies. As shown in our analyses, the timing of GOC discussions matters, with conversations occurring in the hospital likely having a more immediate impact. There remain important barriers to the provision of end-of-life care for patients with hematologic malignancies that need to be addressed.

Prognostic uncertainty, oncology culture, and health system issues represent potentially modifiable barriers to quality end-of-life care. In a 2016 national survey, hematologic oncologists reported that unrealistic patient expectations (97%), clinician concerns about taking away hope (71%), and unrealistic clinical expectations (59%) were key barriers to quality EOL care [32,33]. Reducing prognostic uncertainty may be helpful; several groups have identified prognostic factors or developed prognostic models specifically in patients with hematologic malignancies, although it remains unclear if they have high enough accuracy to guide treatment decisions [34,35]. Oncologists have a critical role defining care decisions in the last weeks of life. In a case vignette study, we previously found that hematologic oncology specialists were much more likely than solid tumor oncology specialists (*p* < 0.0001) to recommend chemotherapy to a patient with performance status of 4 and predicted survival of 1 month, despite controlling for tumor response (15%) and treatment toxicity (moderate) [6]. Hematologic specialists were also more likely to report feeling a sense of failure when they were not able to alter the course of disease (46% vs. 31%, *p* = 0.04) [6]. More cancer treatment at the end of life is associated with increased ICU use [36]. Health system barriers such as the lack of acute palliative care units at cancer centers, limited exposure to palliative care for hematologic oncology trainees, and challenges in arranging transfusions while under hospice may also contribute to higher rates of ICU and hospital death [37,38,39,40].

This study has several limitations. First, it was conducted at a single comprehensive cancer center. The patient characteristics and clinical practice may not apply to other settings. Second, we were only able to use a pre-post design to examine the change in hospital outcomes. Thus, confounders including time and COVID-disease-related changes may contribute to the observed changes. Given the complex and time-sensitive nature of our intervention, it was not possible to conduct a randomized controlled trial. However, this study included consecutive patients and the solid tumor group in this analysis served as a concurrent control. Third, we were not able to retrieve some important variables in this institutional database study, such as disease status, prior treatments, and performance status. In a sensitivity analysis adjusting for chance of recovery from ICU, patients with hematologic malignancies remained at an elevated risk of ICU death. Fourth, we only focused on hospital outcomes and did not collect data on other quality of EOL care metrics, such as chemotherapy use and hospice use. Further studies are needed to assess these outcomes. 

## 5. Conclusions

We identified the differential impact of GOC discussions on hospital outcomes between patients with solid tumors and hematologic malignancies. Our findings highlight that GOC discussions were likely necessary but insufficient to improve hospital outcomes, highlighting opportunities to overcome other barriers to EOL care. Further efforts in education and research may enable a culture shift in hematologic oncology and health system changes towards improved outcomes.

## Figures and Tables

**Table 1:** Patient characteristics.

	Medical Patients (Including ICU)				ICU Patients			
	Solid Tumors		Hematologic Malignancies		Solid Tumors		Hematologic Malignancies	
	2019	2020	2019	2020	2019	2020	2019	2020
Variables	% (SD) *	% (SD) *	% (SD) *	% (SD) *	% (SD) *	% (SD) *	% (SD) *	% (SD) *
Effective Sample Size, n	3577	2986	2663	2373	361	328	305	259
Age, mean (SD)	61.1 (13.7)	61 (13.9)	60.4 (15.6)	60.5 (15.8)	60.4 (14.2)	60.9 (13.6)	61.2 (15.6)	60.9 (15.5)
Sex								
Male	56.2 (49.6)	51.3 (50)	60.6 (48.9)	59.4 (49.1)	59.6 (49.1)	50.9 (50)	63 (48.3)	59.8 (49)
Female	43.8 (49.6)	48.7 (50)	39.4 (48.9)	40.6 (49.1)	40.4 (49.1)	49.1 (50)	37 (48.3)	40.2 (49)
Race								
Black	13.3 (33.9)	15.2 (35.9)	11.1 (31.4)	11.5 (31.9)	16.1 (36.7)	19.2 (39.4)	15.1 (35.8)	15.1 (35.8)
Asian	6 (23.8)	6 (23.7)	4.7 (21.2)	3.8 (19.1)	6.6 (24.9)	6.1 (23.9)	2.6 (16)	5.8 (23.4)
Hispanic	14.9 (35.6)	17.7 (38.2)	17.2 (37.7)	18 (38.4)	16.1 (36.7)	16.8 (37.4)	17.4 (37.9)	20.5 (40.3)
White	63.3 (48.2)	58.4 (49.3)	63.4 (48.2)	64.1 (48)	58.2 (49.3)	54.6 (49.8)	61.3 (48.7)	54.4 (49.8)
Other	2.5 (15.7)	2.6 (16)	3.6 (18.5)	2.7 (16.1)	3 (17.2)	3.4 (18)	3.6 (18.6)	4.2 (20.2)
Cancer Diagnosis								
Bone and articular cartilage	1.6 (12.5)	1.9 (13.7)	0 (0)	0 (0)	1.7 (12.8)	0.3 (5.5)	0 (0)	0 (0)
Breast	9.4 (29.2)	10.6 (30.8)	0 (0)	0 (0)	8.6 (28)	8.5 (27.9)	0 (0)	0 (0)
Digestive organs	30.1 (45.9)	29.5 (45.6)	0 (0)	0 (0)	25.8 (43.7)	26.2 (44)	0 (0)	0 (0)
Eye, brain, and other parts of CNS	2.7 (16.2)	2.9 (16.7)	0 (0)	0 (0)	4.4 (20.6)	3.7 (18.8)	0 (0)	0 (0)
Female genital organs	3.5 (18.3)	4 (19.5)	0 (0)	0 (0)	1.9 (13.8)	2.4 (15.4)	0 (0)	0 (0)
Lip, oral cavity, and pharynx	5.1 (21.9)	5.4 (22.6)	0 (0)	0 (0)	6.4 (24.4)	7.6 (26.5)	0 (0)	0 (0)
Lymphoid, hematopoietic, and related tissue	0 (0)	0 (0)	89.7 (30.4)	89.7 (30.4)	0 (0)	0 (0)	91.5 (27.9)	91.5 (27.9)
Male genital organs	6.6 (24.8)	6.2 (24.1)	0 (0)	0 (0)	3.3 (17.9)	2.4 (15.4)	0 (0)	0 (0)
Melanoma and other malignant neoplasms of skin	3 (17.2)	3 (17.2)	0 (0)	0 (0)	3.9 (19.3)	4 (19.5)	0 (0)	0 (0)
Mesothelial and soft tissue	5.6 (23.1)	5.3 (22.4)	0 (0)	0 (0)	4.7 (21.2)	5.2 (22.2)	0 (0)	0 (0)
Myeloproliferative and myelodysplastic syndromes	0 (0)	0 (0)	10.3 (30.4)	10.3 (30.4)	0 (0)	0 (0)	8.5 (27.9)	8.5 (27.9)
Neuroendocrine tumors	2.6 (15.9)	2.1 (14.3)	0 (0)	0 (0)	3.9 (19.3)	1.8 (13.4)	0 (0)	0 (0)
Respiratory and intrathoracic organs	18.7 (39)	17.2 (37.8)	0 (0)	0 (0)	28 (44.9)	26.5 (44.1)	0 (0)	0 (0)
Thyroid and other endocrine glands	2.2 (14.6)	2.5 (15.5)	0 (0)	0 (0)	1.7 (12.8)	2.1 (14.5)	0 (0)	0 (0)
Urinary tract	8.9 (28.4)	9.4 (29.2)	0 (0)	0 (0)	5.8 (23.4)	9.1 (28.8)	0 (0)	0 (0)
Admission Type								
Elective	8.6 (28)	8.9 (28.5)	30.8 (46.2)	39.2 (48.8)	2.5 (15.6)	3.4 (18)	15.7 (36.4)	15.8 (36.5)
Emergency	58 (49.4)	61.5 (48.7)	41 (49.2)	42.5 (49.4)	69.8 (45.9)	72.9 (44.5)	56.4 (49.6)	69.5 (46)
Urgent	33.5 (47.2)	29.6 (45.6)	28.2 (45)	18.3 (38.7)	27.7 (44.8)	23.8 (42.6)	27.9 (44.8)	14.7 (35.4)
COVID-19 positive	0 (0)	3.5 (18.4)	0 (0)	5.1 (22.1)	0 (0)	0.9 (9.5)	0 (0)	1.5 (12.3)
SOFA Score on admission, mean (SD)	2.6 (2.4)	2.7 (2.4)	3 (2.5)	3.2 (2.7)	4 (3.2)	3.9 (3.1)	5 (3.2)	4.9 (3.4)

Abbreviations: CNS, central nervous system; COVID-19, Coronavirus Disease 2019; ICU, intensive care unit; SOFA, Sequential Organ Failure Assessment. * Unless otherwise specified.

**Table 2:** Differences in hospitalization outcomes before and after myGOC implementation between patients with solid tumors and those with hematologic malignancies.

	Solid Tumors			Hematologic Malignancies			Difference in Change between Heme and Solid Tumors	*p*-Value ^δ^
Outcome	2019	2020	Change between 2019 and 2020	2019	2020	Change between 2019 and 2020		
Died in ICU, % (95% CI)	32.55 (27.69, 37.83)	18.81 (14.8, 23.6)	0.48 * (0.33, 0.69)	26.38 (21.59, 31.81)	28.27 (22.94, 34.29)	1.1 * (0.75, 1.61)	2.29 * (1.35, 3.88)	0.004
ICU length of stay, mean (95% CI)	6.37 (5.59, 7.14)	4.75 (3.93, 5.56)	−1.62 (−2.74, −0.51)	6.9 (6.05, 7.75)	6.09 (5.17, 7.01)	−0.81 (−2.05, 0.43)	0.81 (−0.85, 2.48)	0.34
Overall survival from ICU admission, median (95% CI)	9.35 (7.23, 11.47)	11.86 (8.84, 14.88)	1.27 ^†^ (0.99, 1.63)	12.66 (9.43, 15.89)	10.8 (8.01, 13.6)	0.85 ^†^ (0.65, 1.11)	0.67 ^†^ (0.47, 0.97)	0.10
Number of ICU admissions during same hospital stay, mean (95% CI)	0.089 (0.08, 0.1)	0.093 (0.083, 0.104)	1.04 ^‡^ (0.897, 1.206)	0.112 (0.1, 0.125)	0.108 (0.095, 0.12)	0.96 ǂ (0.82, 1.128)	0.93 ^‡^ (0.74, 1.15)	0.63
ICU hospitalization during hospital admission, % (95% CI)	8.15 (7.3, 9.1)	8.52 (7.58, 9.56)	1.05 * (0.89, 1.24)	9.68 (8.6, 10.89)	9.05 (7.94, 10.29)	0.93 * (0.77, 1.12)	0.88 * (0.69, 1.13)	0.57
Died in hospital, % (95% CI)	6.29 (5.53, 7.15)	4.91 (4.21, 5.72)	0.77 * (0.63, 0.93)	4.67 (3.94, 5.52)	4.93 (4.14, 5.87)	1.06 * (0.83, 1.35)	1.38 * (1.01, 1.88)	0.09
Hospital length of stay, mean (95% CI)	8.81 (8.46, 9.17)	8.62 (8.23, 9)	−0.2 (−0.71, 0.32)	12.01 (11.6, 12.41)	12.47 (12.03, 12.91)	0.46 (−0.13, 1.05)	0.66 (−0.12, 1.44)	0.19

Abbreviations: CI, confidence interval; ICU, intensive care unit; IQR, interquartile range; NR, not reached; SD, standard deviation. * Odds ratio (95% confidence interval). ^†^ Survival time ratio (95% confidence interval). ^‡^ Ratio (95% confidence interval). ^δ^ Hommel-adjusted *p*-values to compensate for multiple comparisons.

**Table 3:** Goals-of-care documentation before vs. after myGOC implementation.

	Solid Tumors			Hematologic Malignancies			Difference in Change between Heme and Solid Tumors	*p*-Value ^†^
Goals-of-Care Documentation	2019	2020	Change between 2019 and 2020	2019	2020	Change between 2019 and 2020		
DNR order during index hospitalization, % (95% CI)	15.95 (14.75, 17.22)	21.57 (20.07, 23.15)	1.45 * (1.29, 1.63)	7.36 (6.46, 8.39)	11.86 (10.6, 13.25)	1.69 * (1.41, 2.03)	1.17 * (0.94, 1.45)	0.17
Interval between DNR order and index admission, mean (95% CI), days	5.68 (4.9, 6.45)	3.66 (2.93, 4.39)	−2.02 (−3.07, −0.97)	16.6 (15.26, 17.94)	9.53 (8.36, 10.69)	−7.08 (−8.81, −5.35)	−5.06 (−7.08, −3.04)	<0.0001
ACP note present during index hospitalization, % (95% CI)	14.87 (13.75, 16.06)	47.96 (46.12, 49.8)	5.28 * (4.7, 5.92)	4.88 (4.13, 5.76)	52.55 (50.42, 54.67)	21.59 * (17.78, 26.21)	4.09 * (3.27, 5.12)	<0.0001
Interval between ACP note documentation and index admission (for patients with ACP during index hospitalization, mean (95% CI), days	4.72 (4.22, 5.22)	1.94 (1.62, 2.25)	−2.78 (−3.37, −2.2)	14.19 (13.15, 15.23)	2.26 (1.9, 2.62)	−11.93 (−13.03, −10.83)	−9.15 (−10.39, −7.91)	<0.0001
MPOA available before or up until end of index hospitalization, % (95% CI)	21.16 (19.82, 22.55)	22.18 (20.7, 23.74)	1.06 * (0.94, 1.2)	22.7 (21.12, 24.36)	24.29 (22.53, 26.13)	1.09 * (0.96, 1.25)	1.03 * (0.86, 1.23)	0.76
Interval between first MPOA document and index admission discharge, mean (95% CI), days	−188.38 (−233.05, −143.72)	−247.85 (−296.6, −199.1)	−59.47 (−124.61, 5.66)	−293.39 (−343.34, −243.45)	−369.78 (−424.15, −315.4)	−76.38 (−148.9, −3.86)	−16.91 (−114.21, 80.39)	0.73
LW available before or up until end of index hospitalization, % (95% CI)	15.47 (14.29, 16.72)	13.51 (12.31, 14.81)	0.85 * (0.74, 0.98)	16.25 (14.88, 17.72)	16.21 (14.74, 17.8)	1.00 * (0.86, 1.16)	1.17 * (0.95, 1.43)	0.27
Interval between first LW document and index admission discharge, mean (95% CI), days	−173.89 (−224.29, −123.49)	−210.56 (−270.12, −151)	−36.67 (−113.49, 40.14)	−314.67 (−370.37, −258.97)	−448.11 (−510.18, −386.04)	−133.44 (−215.42, −51.46)	−96.77 (−208.86, 15.32)	0.18
OOHDNR available before or up until end of index hospitalization, % (95% CI)	5.2 (4.51, 5.98)	6.53 (5.69, 7.49)	1.28 * (1.06, 1.54)	1.22 (0.88, 1.68)	2.24 (1.73, 2.88)	1.85 * (1.23, 2.79)	1.45 * (0.93, 2.28)	0.10
Interval between first OOHDNR document date and index admission discharge date, mean (95% CI), days	77.25 (61.19, 93.31)	30.39 (13.6, 47.19)	−46.86 (−69.89, −23.83)	169.92 (140.16, 199.69)	72.48 (41.38, 103.58)	−97.44 (−139.16, −55.72)	−50.58 (−98.17, −2.99)	0.037

Abbreviations: ACP, advance care planning; CI, confidence interval; DNR, do-not-resuscitate; ICU, intensive care unit; LW, living will; MPOA, medical power of attorney; OOHDNR, out-of-hospital do-not-resuscitate. * Odds ratio (95% CI). ^†^ Hommel-adjusted *p*-values to compensate for multiple comparisons.

**Table 4:** ICU mortality rate by ACP completion status and tumor type.

ICU Mortality	ICU Mortality in 2019 % (95% CI)	ICU Mortality in 2020 % (95% CI)	Change between 2019 and 2020 *	*p*-Value ^†^
Solid tumors				
No ACP by the end of index hospitalization	21.62 (16.26, 28.17)	5.36 (1.25, 20.22)	0.21 (0.04, 0.95)	0.17
ACP note completed before index hospitalization	31.79 (17.47, 50.64)	11.11 (6.01, 19.64)	0.27 (0.1, 0.75)	0.06
ACP note completed during index hospitalization	48.53 (39.05, 58.11)	22.59 (17.09, 29.24)	0.31 (0.18, 0.52)	<0.0001
Hematologic malignancies				
No ACP by the end of index hospitalization	19.45 (14.77, 25.18)	38.52 (15.72, 67.8)	2.59 (0.73, 9.16)	0.55
ACP note completed before index hospitalization	22.94 (2.9, 74.78)	31.64 (22.47, 42.5)	1.55 (0.15, 16.17)	>0.99
ACP note completed during index hospitalization	55.65 (42.62, 67.94)	27.87 (21.19, 35.7)	0.31 (0.16, 0.58)	0.002

Abbreviations: ACP, advance care planning CI, confidence interval; ICU, intensive care unit. * Odds ratio (95% CI). ^†^ Hommel-adjusted *p*-values to compensate for multiple comparisons.

**Table 5:** Sensitivity analysis accounting for prognosis among patients with solid and hematologic malignancies admitted to ICU in 2020.

ICU Outcome	Solid Tumors	Hematologic Malignancies	Difference in Change between Heme and Solid Tumors	*p*-Value *
Died in ICU, % (95% CI)	13.72 (10.07, 18.42)	32.7 (26.41, 39.68)	3.05 (1.88, 4.98)	<0.0001
ICU length of stay, mean (95% CI)	4.78 (4.09, 5.48)	6.15 (5.37, 6.93)	1.37 (0.27, 2.47)	0.015
Overall survival from ICU admission, median (95% CI)	17.54 (10.28, 24.81)	10.98 (7.24, 14.73)	0.63 (0.47, 0.84)	0.002
Number of ICU admissions during same hospital stay, mean (95% CI)	1.09 (0.97, 1.22)	1.19 (1.05, 1.34)	1.09 (0.92, 1.3)	0.31

Abbreviations: CI, confidence interval; ICU, intensive care unit. * Hommel-adjusted *p*-values to compensate for multiple comparisons.

## Data Availability

Individual participant data that underlie the results reported in this article, after deidentification, are available immediately and ending 5 years following publication to investigators whose proposed use of the data has been approved by the institutional review broad at MD Anderson Cancer Centre to achieve aims in the approved proposal. Proposals should be directed to the corresponding author. To gain access, data requestors will need to sign a data access agreement and provide funding support for data retrieval.

## Supplementary Materials

The following supporting information can be downloaded at: https://www.mdpi.com/article/10.3390/cancers15051507/s1, Figure S1: Overall survival from ICU Admission by Cancer Diagnosis and Time Period.
