# Supplementary material for: Differential Impact of a Multicomponent Goals-of-Care Program in Patients with Hematologic and Solid Malignancies
Source: Cancers (Basel). 2023 Feb 28;15(5):1507. doi: 10.3390/cancers15051507 (PMC10001115; doi:10.3390/cancers15051507)
Supplement: Supplementary file 1 [file cancers-15-01507-s001.zip › cancers-2206083-supplementary.pdf]

Supplementary Material

Figure

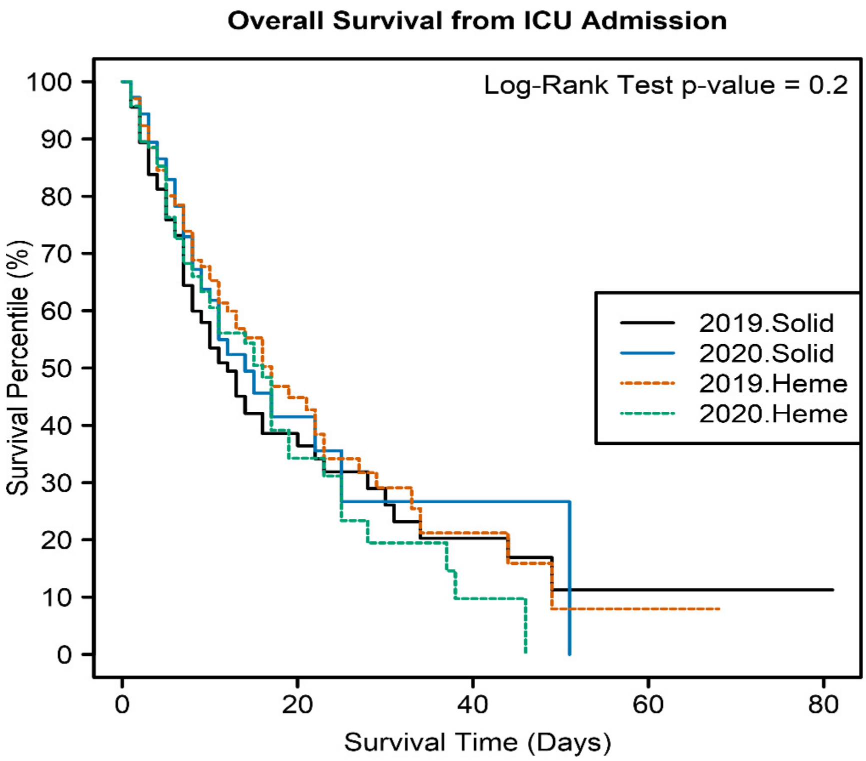

Figure S1. Overall survival from ICU Admission by Cancer Diagnosis and Time Period.
